# *N*-(4-Chloro­phen­yl)-4-ethyl­piperazine-1-carboxamide

**DOI:** 10.1107/S1600536811035331

**Published:** 2011-09-14

**Authors:** Yu-Feng Li

**Affiliations:** a Microscale Science Institute, Department of Chemistry and Chemical Engineering, Weifang University, Weifang 261061, People’s Republic of China

## Abstract

In the title mol­ecule, C_13_H_18_ClN_3_O, the piperazine ring has a chair conformation. In the crystal, mol­ecules are linked into chains along [100] by N—H⋯O hydrogen bonds.

## Related literature

For applications of carboxamide compounds, see: Arrieta *et al.* (2007 ▶). For a related structure, see: Li (2011 ▶).
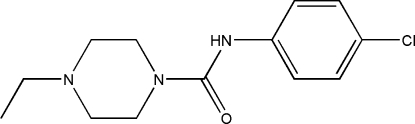


## Experimental

### 

#### Crystal data

C_13_H_18_ClN_3_O
*M* *_r_* = 267.75
Orthorhombic, 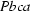

*a* = 9.5546 (19) Å
*b* = 10.910 (2) Å
*c* = 26.477 (5) Å
*V* = 2760.1 (10) Å^3^
*Z* = 8
Mo *K*α radiation
μ = 0.27 mm^−1^
*T* = 293 K
0.25 × 0.22 × 0.21 mm

#### Data collection

Bruker SMART CCD diffractometer
24955 measured reflections
3167 independent reflections
1720 reflections with *I* > 2σ(*I*)
*R*_int_ = 0.079

#### Refinement

*R*[*F* ^2^ > 2σ(*F* ^2^)] = 0.061
*wR*(*F* ^2^) = 0.187
*S* = 1.00
3167 reflections
168 parameters
H atoms treated by a mixture of independent and constrained refinement
Δρ_max_ = 0.27 e Å^−3^
Δρ_min_ = −0.27 e Å^−3^

### 

Data collection: *SMART* (Bruker, 1997 ▶); cell refinement: *SAINT* (Bruker, 1997 ▶); data reduction: *SAINT*; program(s) used to solve structure: *SHELXS97* (Sheldrick, 2008 ▶); program(s) used to refine structure: *SHELXL97* (Sheldrick, 2008 ▶); molecular graphics: *SHELXTL* (Sheldrick, 2008 ▶); software used to prepare material for publication: *SHELXTL*.

## Supplementary Material

Crystal structure: contains datablock(s) global, I. DOI: 10.1107/S1600536811035331/lh5326sup1.cif

Structure factors: contains datablock(s) I. DOI: 10.1107/S1600536811035331/lh5326Isup2.hkl

Additional supplementary materials:  crystallographic information; 3D view; checkCIF report

## Figures and Tables

**Table 1:** Hydrogen-bond geometry (Å, °)

*D*—H⋯*A*	*D*—H	H⋯*A*	*D*⋯*A*	*D*—H⋯*A*
N3—H3*A*⋯O1^i^	0.82 (3)	2.18 (3)	2.986 (3)	167 (2)

Symmetry code: (i) 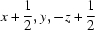.

**Fig. 1.**
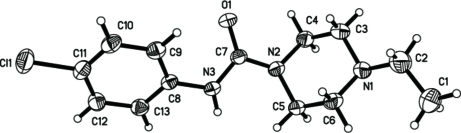
The molecular structure of the title compound showing 30% probability displacement ellipsoids.

## supplementary crystallographic information

### Comment

Carboxamide compounds are an important intermediate reagent in organic synthesis (Arrieta *et al.*, 2007). The molecular structure of the title compound is shown in Fig. 1. The piperazine ring (N1/N2/C3-C6) is in a chair conformation. Bond lengths and angles are comparable to those common to a similar structure (Li, 2011).

### Experimental

A mixture of 1-ethylpiperazine (0.1 mol), and (4-chlorophenyl)carbamic chloride (0.1 mol) was stirred in refluxing ethanol (20 ml) for 4 h to afford the title compound (0.065 mol, yield 65%). Colourless blocks were obtained by recrystallization of a solution of the title compound in ethanol at room temperature.

### Refinement

H atoms boned to C atoms were fixed geometrically and allowed to ride on their attached atoms, with C—H distances = 0.93–0.97 Å and with *U*_iso_(H) = 1.2*U*_eq_(C) or 1.5*U*_eq_(C_methyl_). The N—H hydrogen was refined independently with an isotropic displacement parameter.

### Crystal data

**Table:**

C_13_H_18_ClN_3_O	*F*(000) = 1136
*M**_r_* = 267.75	*D*_x_ = 1.289 Mg m^−^^3^
Orthorhombic, *P**b**c**a*	Mo *K*α radiation, λ = 0.71073 Å
Hall symbol: -P 2ac 2ab	Cell parameters from 1720 reflections
*a* = 9.5546 (19) Å	θ = 3.2–27.2°
*b* = 10.910 (2) Å	µ = 0.27 mm^−^^1^
*c* = 26.477 (5) Å	*T* = 293 K
*V* = 2760.1 (10) Å^3^	Block, colorless
*Z* = 8	0.25 × 0.22 × 0.21 mm

### Data collection

**Table:**

Bruker SMART CCD diffractometer	1720 reflections with *I* > 2σ(*I*)
Radiation source: fine-focus sealed tube	*R*_int_ = 0.079
graphite	θ_max_ = 27.5°, θ_min_ = 3.1°
φ and ω scans	*h* = −11→12
24955 measured reflections	*k* = −14→14
3167 independent reflections	*l* = −34→33

### Refinement

**Table:**

Refinement on *F*^2^	Secondary atom site location: difference Fourier map
Least-squares matrix: full	Hydrogen site location: inferred from neighbouring sites
*R*[*F*^2^ > 2σ(*F*^2^)] = 0.061	H atoms treated by a mixture of independent and constrained refinement
*wR*(*F*^2^) = 0.187	*w* = 1/[σ^2^(*F*_o_^2^) + (0.1045*P*)^2^] where *P* = (*F*_o_^2^ + 2*F*_c_^2^)/3
*S* = 1.00	(Δ/σ)_max_ < 0.001
3167 reflections	Δρ_max_ = 0.27 e Å^−^^3^
168 parameters	Δρ_min_ = −0.27 e Å^−^^3^
0 restraints	Extinction correction: *SHELXL97* (Sheldrick, 2008), Fc^*^=kFc[1+0.001xFc^2^λ^3^/sin(2θ)]^-1/4^
Primary atom site location: structure-invariant direct methods	Extinction coefficient: 0.021 (3)

### Special details

**Table:**

Geometry. All e.s.d.'s (except the e.s.d. in the dihedral angle between two l.s. planes) are estimated using the full covariance matrix. The cell e.s.d.'s are taken into account individually in the estimation of e.s.d.'s in distances, angles and torsion angles; correlations between e.s.d.'s in cell parameters are only used when they are defined by crystal symmetry. An approximate (isotropic) treatment of cell e.s.d.'s is used for estimating e.s.d.'s involving l.s. planes.
Refinement. Refinement of *F*^2^ against ALL reflections. The weighted *R*-factor *wR* and goodness of fit *S* are based on *F*^2^, conventional *R*-factors *R* are based on *F*, with *F* set to zero for negative *F*^2^. The threshold expression of *F*^2^ > σ(*F*^2^) is used only for calculating *R*-factors(gt) *etc*. and is not relevant to the choice of reflections for refinement. *R*-factors based on *F*^2^ are statistically about twice as large as those based on *F*, and *R*- factors based on ALL data will be even larger.

### Fractional atomic coordinates and isotropic or equivalent isotropic displacement parameters (Å^2^)

**Table:**

	*x*	*y*	*z*	*U*_iso_*/*U*_eq_	
Cl1	0.12528 (9)	0.54490 (8)	0.41931 (3)	0.0847 (4)	
O1	0.14283 (15)	0.18919 (18)	0.21930 (6)	0.0616 (5)	
N3	0.3536 (2)	0.2579 (2)	0.24861 (7)	0.0514 (6)	
C8	0.2986 (2)	0.3286 (2)	0.28864 (8)	0.0458 (6)	
N2	0.34171 (19)	0.1241 (2)	0.18146 (8)	0.0580 (6)	
C13	0.3594 (2)	0.3193 (2)	0.33612 (9)	0.0542 (6)	
H13A	0.4350	0.2671	0.3412	0.065*	
N1	0.4083 (2)	0.1241 (2)	0.07662 (7)	0.0644 (7)	
C11	0.1940 (3)	0.4637 (2)	0.36844 (9)	0.0567 (7)	
C12	0.3070 (3)	0.3881 (3)	0.37613 (9)	0.0596 (7)	
H12A	0.3482	0.3829	0.4079	0.072*	
C10	0.1353 (3)	0.4758 (3)	0.32123 (10)	0.0590 (7)	
H10A	0.0605	0.5290	0.3163	0.071*	
C7	0.2716 (2)	0.1909 (2)	0.21612 (8)	0.0483 (6)	
C9	0.1878 (2)	0.4087 (2)	0.28136 (9)	0.0547 (6)	
H9A	0.1486	0.4173	0.2494	0.066*	
C6	0.4827 (3)	0.2016 (3)	0.11215 (10)	0.0652 (7)	
H6A	0.4382	0.2815	0.1134	0.078*	
H6B	0.5782	0.2129	0.1006	0.078*	
C4	0.2660 (3)	0.0469 (3)	0.14571 (9)	0.0639 (7)	
H4A	0.1702	0.0367	0.1571	0.077*	
H4B	0.3094	−0.0334	0.1443	0.077*	
C5	0.4841 (2)	0.1467 (3)	0.16426 (9)	0.0612 (7)	
H5A	0.5360	0.0703	0.1638	0.073*	
H5B	0.5305	0.2023	0.1874	0.073*	
C3	0.2667 (3)	0.1030 (3)	0.09430 (10)	0.0707 (8)	
H3B	0.2187	0.0490	0.0709	0.085*	
H3C	0.2164	0.1802	0.0952	0.085*	
C2	0.4049 (4)	0.1795 (4)	0.02561 (12)	0.0997 (12)	
H2A	0.3649	0.2610	0.0280	0.120*	
H2B	0.3440	0.1308	0.0042	0.120*	
C1	0.5445 (4)	0.1884 (5)	0.00140 (12)	0.1227 (16)	
H1A	0.5349	0.2248	−0.0314	0.184*	
H1B	0.5840	0.1079	−0.0019	0.184*	
H1C	0.6048	0.2382	0.0219	0.184*	
H3A	0.435 (3)	0.236 (2)	0.2526 (8)	0.053 (7)*	

### Atomic displacement parameters (Å^2^)

**Table:**

	*U*^11^	*U*^22^	*U*^33^	*U*^12^	*U*^13^	*U*^23^
Cl1	0.0886 (6)	0.0831 (6)	0.0824 (6)	0.0078 (4)	0.0226 (4)	−0.0221 (4)
O1	0.0337 (8)	0.0846 (14)	0.0665 (11)	−0.0008 (8)	0.0028 (7)	−0.0060 (9)
N3	0.0335 (10)	0.0646 (14)	0.0561 (12)	0.0059 (9)	−0.0007 (8)	−0.0081 (10)
C8	0.0382 (11)	0.0471 (14)	0.0521 (13)	−0.0021 (10)	0.0039 (9)	0.0014 (10)
N2	0.0406 (10)	0.0713 (16)	0.0622 (13)	−0.0072 (10)	0.0060 (9)	−0.0169 (11)
C13	0.0484 (13)	0.0530 (16)	0.0613 (15)	0.0056 (11)	−0.0028 (11)	0.0003 (12)
N1	0.0520 (12)	0.0864 (18)	0.0547 (12)	−0.0125 (12)	−0.0010 (9)	−0.0015 (11)
C11	0.0547 (14)	0.0511 (16)	0.0643 (15)	−0.0002 (12)	0.0145 (12)	−0.0034 (12)
C12	0.0600 (15)	0.0637 (18)	0.0552 (14)	−0.0045 (13)	0.0019 (11)	−0.0056 (12)
C10	0.0504 (14)	0.0499 (16)	0.0766 (17)	0.0083 (11)	0.0075 (12)	0.0008 (13)
C7	0.0386 (12)	0.0567 (15)	0.0496 (12)	0.0009 (11)	0.0008 (10)	0.0032 (11)
C9	0.0488 (13)	0.0554 (16)	0.0597 (14)	0.0032 (12)	0.0002 (11)	0.0063 (12)
C6	0.0466 (13)	0.075 (2)	0.0737 (16)	−0.0132 (13)	0.0069 (12)	−0.0094 (15)
C4	0.0510 (14)	0.075 (2)	0.0658 (15)	−0.0186 (13)	0.0059 (12)	−0.0164 (13)
C5	0.0380 (12)	0.084 (2)	0.0618 (15)	0.0002 (12)	−0.0005 (11)	−0.0181 (13)
C3	0.0469 (14)	0.096 (2)	0.0691 (17)	−0.0125 (15)	−0.0036 (12)	−0.0090 (16)
C2	0.083 (2)	0.144 (4)	0.072 (2)	−0.015 (2)	−0.0036 (17)	0.023 (2)
C1	0.097 (3)	0.194 (5)	0.077 (2)	−0.033 (3)	0.0140 (19)	0.021 (2)

### Geometric parameters (Å, °)

**Table:**

Cl1—C11	1.741 (2)	C10—H10A	0.9300
O1—C7	1.233 (3)	C9—H9A	0.9300
N3—C7	1.374 (3)	C6—C5	1.504 (4)
N3—C8	1.413 (3)	C6—H6A	0.9700
N3—H3A	0.82 (3)	C6—H6B	0.9700
C8—C9	1.386 (3)	C4—C3	1.492 (4)
C8—C13	1.389 (3)	C4—H4A	0.9700
N2—C7	1.350 (3)	C4—H4B	0.9700
N2—C5	1.456 (3)	C5—H5A	0.9700
N2—C4	1.458 (3)	C5—H5B	0.9700
C13—C12	1.391 (3)	C3—H3B	0.9700
C13—H13A	0.9300	C3—H3C	0.9700
N1—C3	1.450 (3)	C2—C1	1.482 (5)
N1—C6	1.451 (3)	C2—H2A	0.9700
N1—C2	1.480 (4)	C2—H2B	0.9700
C11—C12	1.374 (4)	C1—H1A	0.9600
C11—C10	1.377 (3)	C1—H1B	0.9600
C12—H12A	0.9300	C1—H1C	0.9600
C10—C9	1.379 (3)		
			
C7—N3—C8	123.20 (19)	N1—C6—H6B	109.3
C7—N3—H3A	117.5 (17)	C5—C6—H6B	109.3
C8—N3—H3A	114.8 (16)	H6A—C6—H6B	108.0
C9—C8—C13	119.4 (2)	N2—C4—C3	110.7 (2)
C9—C8—N3	121.6 (2)	N2—C4—H4A	109.5
C13—C8—N3	118.9 (2)	C3—C4—H4A	109.5
C7—N2—C5	125.8 (2)	N2—C4—H4B	109.5
C7—N2—C4	120.42 (19)	C3—C4—H4B	109.5
C5—N2—C4	111.01 (18)	H4A—C4—H4B	108.1
C8—C13—C12	120.0 (2)	N2—C5—C6	110.24 (19)
C8—C13—H13A	120.0	N2—C5—H5A	109.6
C12—C13—H13A	120.0	C6—C5—H5A	109.6
C3—N1—C6	109.9 (2)	N2—C5—H5B	109.6
C3—N1—C2	109.8 (2)	C6—C5—H5B	109.6
C6—N1—C2	111.4 (2)	H5A—C5—H5B	108.1
C12—C11—C10	120.9 (2)	N1—C3—C4	111.3 (2)
C12—C11—Cl1	119.17 (19)	N1—C3—H3B	109.4
C10—C11—Cl1	120.0 (2)	C4—C3—H3B	109.4
C11—C12—C13	119.6 (2)	N1—C3—H3C	109.4
C11—C12—H12A	120.2	C4—C3—H3C	109.4
C13—C12—H12A	120.2	H3B—C3—H3C	108.0
C11—C10—C9	119.7 (2)	N1—C2—C1	113.7 (3)
C11—C10—H10A	120.1	N1—C2—H2A	108.8
C9—C10—H10A	120.1	C1—C2—H2A	108.8
O1—C7—N2	122.2 (2)	N1—C2—H2B	108.8
O1—C7—N3	122.3 (2)	C1—C2—H2B	108.8
N2—C7—N3	115.43 (19)	H2A—C2—H2B	107.7
C10—C9—C8	120.4 (2)	C2—C1—H1A	109.5
C10—C9—H9A	119.8	C2—C1—H1B	109.5
C8—C9—H9A	119.8	H1A—C1—H1B	109.5
N1—C6—C5	111.5 (2)	C2—C1—H1C	109.5
N1—C6—H6A	109.3	H1A—C1—H1C	109.5
C5—C6—H6A	109.3	H1B—C1—H1C	109.5

### Hydrogen-bond geometry (Å, °)

**Table:**

*D*—H···*A*	*D*—H	H···*A*	*D*···*A*	*D*—H···*A*
N3—H3A···O1^i^	0.82 (3)	2.18 (3)	2.986 (3)	167 (2)

Symmetry codes: (i) *x*+1/2, *y*, −*z*+1/2.
